# Stereotactic Body Radiation Therapy (SBRT) in prostate cancer in the presence of hip prosthesis – is it a contraindication? A narrative review

**DOI:** 10.1186/s12894-024-01479-8

**Published:** 2024-07-26

**Authors:** Sheen Dube, Vibhay Pareek, Mansi Barthwal, Febin Antony, David Sasaki, Ryan Rivest

**Affiliations:** 1 Department of Biochemistry, University of Winnipeg, Winnipeg, MB Canada; 2 Dept. of Radiation Oncology, CancerCare Manitoba, 675 McDermot Ave, Winnipeg, Winnipeg, MB, MB R3E 0V9 Canada; 3 Department of Medical Physics, CancerCare Manitoba, Winnipeg, MB Canada

## Abstract

Hip replacement is a common orthopedic surgery in the aging population. With the rising incidence of prostate cancer, metallic hip prosthetics can cause considerable beam hardening and streak artifacts, leading to difficulty in identifying the target volumes and planning process for radiation treatment. The growing use of Stereotactic Body Radiation Therapy (SBRT) to treat prostate cancer is now well established. However, the use of this treatment modality in the presence of a hip prosthesis is poorly understood. There is enough literature on planning for external beam radiation treatment without any difficulties in the presence of hip prosthesis with conventional or Hypofractionated treatment. However, there is a shortage of literature on the impact of the prosthesis in SBRT planning, and there is a need for further understanding and measures to mitigate the obstacles in planning for SBRT in the presence of hip prosthesis. We present our review of the intricacies that need to be understood while considering SBRT in the presence of hip prostheses in prostate cancer treatment.

## Introduction

Stereotactic Body Radiation Therapy (SBRT) has evolved **significantly over the last two decades** in the management of prostate cancer [1]. However, there are scenarios where this method of treatment delivery is contraindicated [2]. The presence of hip implants has been considered a hindrance in treatment planning and sometimes can be viewed as a relative contraindication for consideration of SBRT treatment. Considering the age demographics of prostate cancer, hip replacements are prevalent in the elderly patient population [3], leading to challenges in delivering the **adequate** radiation treatment for prostate cancer. There has also been a note in the increase in the number of hip replacements being done over the years [4] from 210 per 100 000 males in the year 1998–1999 to the rise of up to 265 cases undergoing the implant in 2006–2007. The computed tomography (CT) imaging data are affected by the metallic implants due to the extensive range of Hounsfield units (HU) associated with erroneous CT values and cause artifacts in the CT images for radiation treatment planning [5].

The artifacts caused by the prostheses interfere with the contouring of the target and organs, and the implants also complicate the radiation planning process due to incorrectly modeled radiation dose scattering and dose attenuation, leading to dose uncertainty [6]. Similar problems have been encountered in conventional planning for external beam radiation therapy with IMRT and VMAT techniques [7]. However, there have been measures to overcome the same. The American Association of Physicists in Medicine Task Group (AAPM-TG) report 63 has provided a few solutions for managing treatment planning with the hip implant in place [6]. Also, it **is not** easy to assess the prosthesis electron density accurately. Thus, the planning systems find it difficult to accurately model the dose in, near, and beyond the implant for the megavoltage photon beam placed [6].

With the 10-MV treatment beam, the attenuation occurring due to the titanium hip implant is about 2.5 times larger than the bone and nearly four times higher than the water-equivalent tissue [8]. Depending on the photon beam energy and hip prosthesis composition, the dose attenuation could range from 10 to 60% [6]. The backscatter increases with the increasing power and the atomic number of the material used for the prosthesis, and it becomes a significant concern at the bone-implant surface interface [9].

With the increasing radiation treatment delivery with SBRT, conventional planning has reduced [10], and hence, these measures need to be reviewed to be employed in moderate to ultra-hypofractionation, including SBRT. The recent CHHip trial also reported hesitancy in treating patients with bilateral hip implants with SBRT [11]. The hip implants are not considered contraindications for CT and MRI simulation or radiation fraction delivery [12]. In this review article, we aim to present the technical aspects associated with planning the SBRT cases with bilateral hip implants and report the accurate and safe approach for prostate radiation treatment in the presence of bilateral hip implants.

## Methodology

We performed a PubMed search with the following MesH terms: Prostate Cancer, Prostate treatment, Prostatic malignancy AND SBRT, SABR, VMAT, IMRT, AND bilateral hip implants, bilateral hip prosthesis, metallic hip implants, along with the articles published in the various oncology journals through google search and the individual websites. **The literature search was focused between January 1990 to June 2022**. The focus was on the best available practice regarding radiation therapy delivery in institutes that addressed the treatment planning and delivery of bilateral hip implants in the patient population with prostate cancer.

## Results and discussion

### Imaging for target delineation/planning

#### Image registration

Due to image artifacts, hip implants can cause problems in defining the clinical target volume (CTV) of the prostate and the organs at risk, including the bladder and the rectum. The role of MRI in such scenarios is valuable, and the combined CT-MR image registration helps define the target and the organs at risk better [12]. The CT and MR images need to be transferred to the treatment planning system for image registration, which is done using at least three bony landmarks, and after that, proceed with planning. Other measures that deal with the problem of target delineation include approximating target volumes from a predicted anatomical position using the available bone landmarks. However, this does not provide the conformality needed for SBRT planning. The CT-MR registration has shown improvement in the target delineation. Studies have shown that CT-derived prostate volumes are more extensive than MR-derived volumes, especially toward the seminal vesicles (by 6 mm) and the apex of the prostate [13]. Similarly, it was noted that the dose-volume histograms from CT and MRI comparison showed we could spare a mean of 10% of rectal volume and approximately 5% of bladder and femoral heads, respectively; thus, the co-registration can help avoid the overestimation of CTV with CT images compared with MRI [14]. The concern lies in the CT-based imaging on the flat couch and the MR images undertaken on a standard MR couch with a firm mattress. However, the difference did not impact the registration and further did not impact the target and organs at-risk delineation [12]. The study also noted on qualitative analysis that the phantom-filled water tubes demonstrated distortions less than 0.2 cm at distances greater than 3 cm from the prosthesis.

#### Metal artifact reduction (MAR) algorithms for CT imaging

**Fig. 1:**
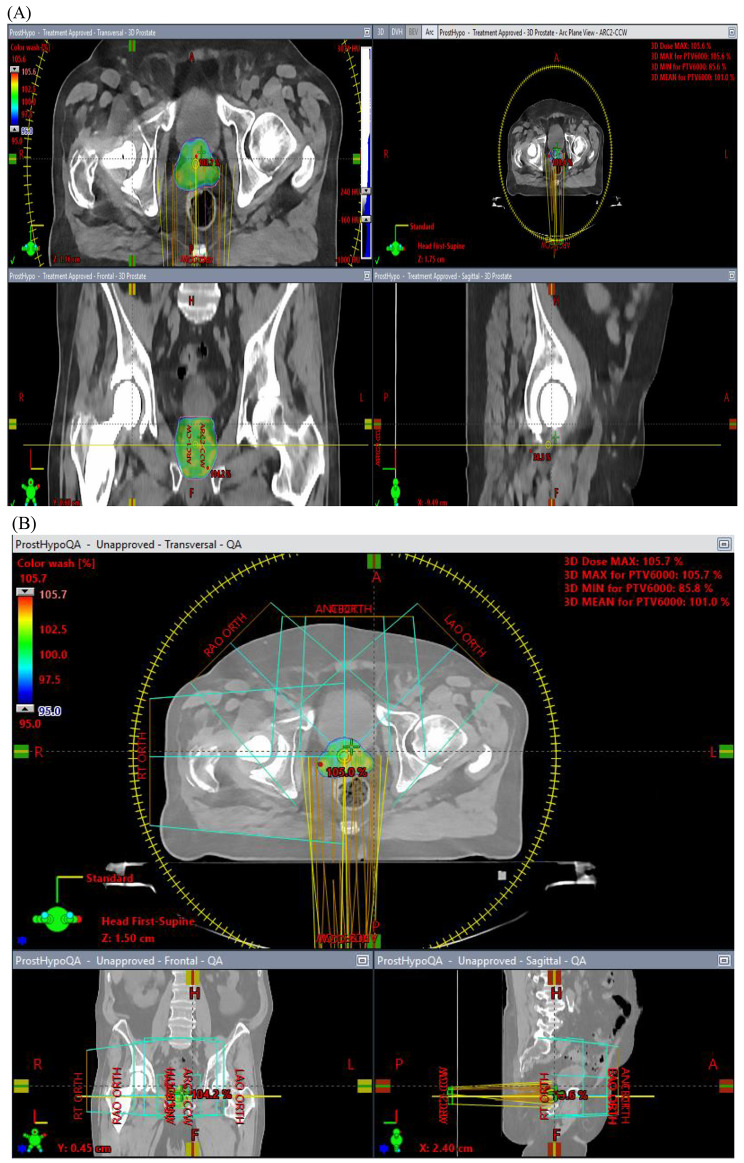
Axial contrast-enhanced CT scans of a 76-year-old man with a right metal hip prosthesis who underwent radiation treatment for intermediate-risk prostate cancer **(A)** and the imaging on quality assurance **(B)**

**Fig. 2:**
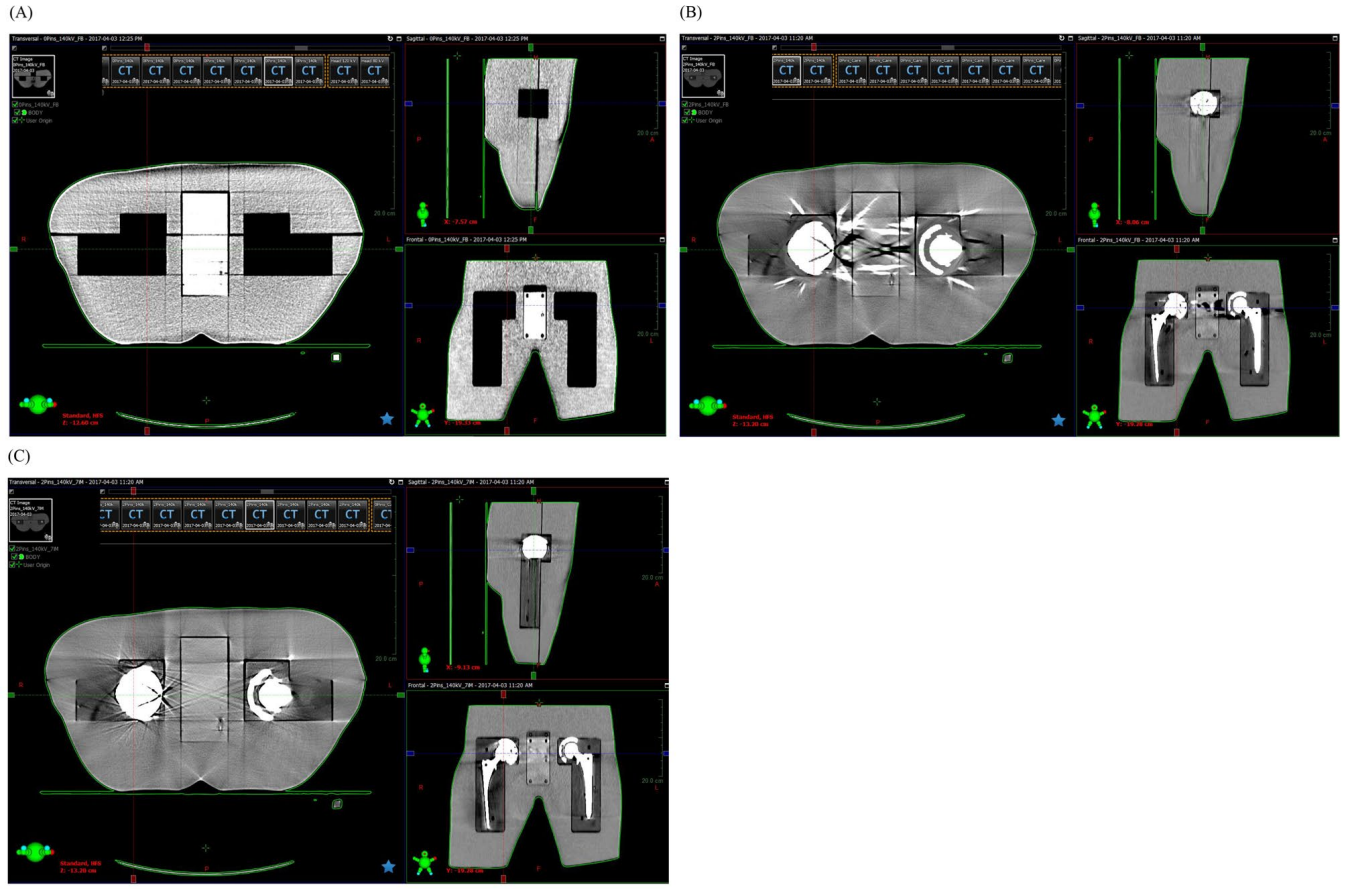
**(A)** standard filtered back projection reconstruction of a phantom with metal implants removed. **(B)** standard filtered back projection reconstruction of a phantom with metal implants in place. **(C)** MAR reconstruction with metal implants in place

Metal artifact reduction (MAR) methods have been extensively reviewed [15], and commercial MAR helps improve treatment planning. It is also easy to use as it requires minimal skills for the operations; for example, the ker-MAR has been shown to have improved efficiency in head and neck cases [16]. On the other hand, the MRI-based CT MAR [17] requires the manual selection of CT scans for artifact correction. Similarly, the deep learning-based algorithms (DL-MAR) do not require paired data for artifact corrections with similar outcomes compared to the water density override. The commercial MAR methods, including O-MAR, iMAR, Smart MAR, and SEMAR, are commonly used and have the advantage of a wide range of clinical applications. However, they have shown incomplete artifact removal with Hounsfield unit errors and may induce new artifacts [18–20]. The density correction methods, though available in the treatment planning system, require expertise and training for artifact identification and density overrides [21, 22]. The deep-learning-based MAR algorithms do not require sinogram or paired data and help reduce the metal artifacts on the CT scans with comparable performance to density correction for dose calculation accuracy. However, the drawback is that it requires paired data and depends upon the NMAR application [23–25]. Considering the advantages and the disadvantages of the MAR method, it appears to be a promising solution for developing these algorithms, which would help target specific patterns of metal artifacts and specific anatomical structures, thus proving beneficial for future radiation treatment applications. The commercially available MAR options are shown in Table 1. Figure 1 shows an axial contrast-enhanced CT scan of a 76-year-old man with a right metal hip prosthesis who underwent radiation treatment for intermediate-risk prostate cancer (A) and the imaging on quality assurance (B). Figure 2 shows (A) Standard filtered back projection reconstruction of a phantom with metal implants removed (B) standard filtered back projection reconstruction of a phantom with metal implants in place (C) MAR reconstruction with metal implants in place. Table 2 summarizes various publication results for imaging for target delineation and planning with hip prostheses.

**Table 1:** Various MAR Applications available for RT Applications

MAR Method	Examples	Comments
Commercial	O-MAR, iMAR, SMART MAR, SEMAR	1. Applicable for various RT clinical cases and applications 2. Incomplete artifact removal with HU errors 3. Scanner specific with manual methods and needs specialized operators
Research-based	Image-based MAR, MDT, Sinogram-based MAR, ALIR, kerMAR, MVCBCT and kvCT method	1. Reduces dose errors and spot artifacts 2. Do not require sinogram and threshold-based tissue classification 3. Long processing times 4. Requires MVCBCT scans leading to addition radiation burden
Deep learning-based	RL-ARCNN and DL-MAR	1. Reduces remaining metal artifacts on CT scans after NMAR application 2. No need for sinogram data 3. NMAR performance dependent 4. Applicability limited to head and neck cases

**Table 2:** Summary of measures and results for imaging for target delineation and planning with hip prostheses

Author	Summary of measures and results
Charnley et al. [12] *N* = 4	• Pelvic CT studies were obtained for each patient for radiotherapy treatment planning • The CT slice thickness and spacing were 5 mm. Following observation of the artifacts demonstrated in • T2 weighted images were acquired and the slice thickness and separation were 5 mm. • All CT and MR images were transferred for image registration and subsequent planning • Identification of at least three anatomical landmarks in each image set • A dosimetrist and a clinical oncologist performed all registration work • Once the minimum number of points has been entered and the registration is active, the operator may enter test points to assess the accuracy achieved and convert them to registration points if acceptable. • A better than 5 mm accuracy was obtained over the clinically relevant volume. • After registration, a CTV was marked on the MR study and transferred to the CT dataset for expansion to a PTV using our standard margins and subsequent conformal 3D treatment • Treatment plans were produced without density correction
Rasch et al. [13] *N* = 18	• Outline the prostate without seminal vesicles both on CT, and axial, coronal, and sagittal MR images • CT and MR scans were matched in three dimensions using matching on bony structures. • The volumes measured and the interscan and interobserver variation determined. • A urethrogram was performed, and the location of the tip of the dye column was compared with the apex delineated in CT and MR images. • The average ratio between the CT and MR volumes was 1.4 (*p* < 0.005). • Only minor differences were observed between the volumes outlined in the various MR scans, although the coronal MR volumes were the smallest. • The CT-derived prostate was 8 mm larger at the base of the seminal vesicles and 6 mm larger at the apex of the prostate than the axial MRI. • CT-derived prostate volumes are larger than MR-derived volumes, especially toward the seminal vesicles and the apex of the prostate. • Using MRI for delineation of the prostate reduces the amount of irradiated rectal wall and could reduce rectal and urological complications.
Sannazzari et al. [14] *N* = 8	• The clinical target volume (CTV) (prostate plus seminal vesicles) was delineated on CT and MRI studies, and image fusion was obtained from the superimposition of anatomical fiducial markers. • For both studies, dose–volume histograms relative to CTV, rectum, bladder, and femoral heads were compared. • Image fusion showed a mean overestimation of CTV of 34% with CT compared with MRI. • Along the anterior–posterior and superior–inferior directions, CTV was a mean 5 mm larger with CT study than MRI. • The dose–volume histograms resulting from CT and MRI comparison showed that it is possible to spare a mean of 10% of rectal volume and approximately 5% of bladder and femoral heads, respectively
Filograna et al. [26] *N* = 20	• Thirty metallic implants in 20 consecutive cadavers with metallic implants underwent both single-energy CT (SECT) and dual-energy CT (DECT) with a clinically suitable scanning protocol. • Extrapolated monoenergetic DECT images were generated at 64, 69, 88, 105, 120, and 130 keV and individually adjusted monoenergy for optimized image quality (OPTkeV). • Qualitative and quantitative analyses showed statistically significant differences between monoenergetic DECT extrapolated images and SECT, with improved diagnostic assessment in monoenergetic DECT at higher moon energies. • This study demonstrates that monoenergetic DECT images extrapolated at high energy levels significantly reduce metallic artifacts from orthopedic implants and improve image quality compared to SECT examination
Guggenberger et al. [27] N = NA	• Five vendors’ posterior spinal fusion implants for the cervical, thoracic, and lumbar spine were examined ex vivo with single-energy (SE) CT (120 kVp) and DECT (140/100 kVp). • Extrapolated monoenergetic DECT images were generated at 64, 69, 88, and 105 keV, and the mono energy was individually adjusted for optimized image quality (OPTkeV). • Monoenergetic DECT provides significantly better image quality and fewer metallic artifacts from implants than SECT. • Using individual keV values for vendor and spine level is recommended.
Yue et al. [28] *N* = 35	• Four sets of VMS images without MARs and four sets of VMS images with MARs were obtained. • Artifact index (AI), CT number, and SD value were assessed at the periprosthetic region and the pelvic organs. • The AIs in 120 and 140 keV images were significantly lower than those in 80 and 100. • The AIs of the periprosthetic region in VMS images with MARs were significantly lower than those in VMS images without MARs. At the same time, the AIs of pelvic organs were not significantly different. • VMS images with MARs improved the accuracy of CT numbers for the periprosthetic region. • VMS images with MARs at 120 and 140 keV had higher subjective scores and could improve the image quality, leading to reliable diagnosis of prosthesis-related problems.
Aubin et al. [29] *N* = 7	• MV CBCT images were imported into the treatment planning system and registered with the original planning CT using bony anatomy contoured on each image set. • The target volumes and organs at risk for prostate treatment were contoured using both the CT and the MV CBCT for single hip replacement and using only the MV CBCT for bi-lateral hip prostheses. • The MV CBCT images could clearly visualize the hip prosthesis and bony anatomy and provide sufficient soft-tissue contrast to help delineate the prostate, bladder, and rectum. • The MV CBCT images were particularly useful in helping delineate these structures as well as the lateral extension of the prostate in the axial plane, the seminal vesicles, and the lymph nodes. • The prostate volumes contoured with the help of MV CBCT were generally smaller than the volumes that would have been estimated using only the regular CT, which contains severe artifacts. • Target delineation for the patient with bilateral hip prostheses was entirely performed using the MV CBCT since the relevant organs were obscured due to the severity of the artifacts on the conventional CT. • MV CBCT registered with the planning CT can complement missing information and facilitate segmentation for planning purposes when hip prostheses are present.

#### Use of multi-energy CT for artifact reduction

The CT systems are equipped with polychromatic X-rays comprising a spectrum of varying energy photons representing the cumulative transmitted intensity at each energy forming the complete beam. Using such beams for longer path lengths and high atomic number materials can cause beam hardening artifacts. When the correction algorithms are unmet, they can lead to shading and dark bands on the planning images [30]. The multi-energy CT scans can help improve beam-hardening correction using virtual monoenergetic images used in CT myocardial perfusion applications [31], and similar improvements in the images have been found in dental restorations [32]. Virtual monoenergetic images help reduce image artifacts. In one of the studies, it was recommended to use the routine reconstruction of the virtual monoenergetic images at 50 keV using the dual-energy CT, and for reducing the beam-hardening artifacts, the virtual monoenergetic imaging at 120 keV was found to be useful [33]. Other studies have shown a similar impact in reducing the metal artifacts, especially with the prosthesis in situ [26–28, 34–36].

#### Treatment planning with Megavoltage CT (MVCT) images of helical tomotherapy

Tomotherapy generates lower energy MVCT images using the same therapeutic megavoltage x-ray beam, and the uniformity and spatial resolution of these MVCT images are comparable to the diagnostic CT images [37]. These MVCT images have poorer noise and low-contrast resolution outcomes than diagnostic CT scanners but produce sufficient contrast to help delineate soft tissue structures. A case study of prostate cancer with bilateral hip implants reported the use of MVCT with helical tomotherapy showed no artifacts in the MVCT, and the same images were used for the planning, and the plan was optimized to the planned constraints [38]. The reported patient image dose using MVCT with helical tomotherapy is 1.5 cGy per scan [39], and the use of MV-CBCT to complement standard CT for target definition in prostate cancers being treated with hip implants has been reported [29].

#### Ultrasound used for target volume definition

Using stereotactic ultrasound for daily prostate image guidance has helped reduce the intrafraction motion during the treatment [40, 41] and define the PTV [42]. A case report utilizing the definition of PTV based on the ultrasound images acquired during the CT simulation and matched to the planning CT and the ultrasound referenced to the isocenter of the CT simulator showed better matching of the prostate, seminal vesicles, and the target volumes [43]. The prosthesis-associated artifacts can be further reduced when added with advanced reconstruction algorithms with artifact suppression [44, 45] and dual-energy CT [46].

### Treatment planning

#### IMRT planning in bilateral hip prosthesis

The reports on using IMRT while planning in the presence of hip implants have employed dosimetric constraints focusing on no dose entering or exiting the prosthesis by using non-coplanar beams [47]. Another study utilized coplanar beams and avoided the prosthesis in the beam’s eye view and found IMRT to be superior in terms of target coverage and reduction in doses to organs at risk with a significant decrease in the rectum dose with composite V80% of 35% for IMRT vs. 70% for the 3D-CRT plan and bladder dose reduced to 9% vs. 20% [48].

The AAPM TG 63 recommends avoiding beams passing through the hip prostheses [6] based on results from previous studies that demonstrated an impact on the dosimetric outcomes [49]. The advantage is that the certainty for the PTV dose is achieved, and there is a possibility to expand the gantry angles for coplanar IMRT beams, leading to improved dose-volume histograms as compared to that without the modification related to the implant, causing the beams to be more constrained to narrow anterior and posterior range [6]. In one case study inverse IMRT planning with geometric constraint instead of the dosimetric constraint was employed [50], defying the usual understanding proposed by AAPM TG 63 to avoid beams passing through hip prostheses that need dosimetric constraints. They concluded that avoiding beam entry alone would provide certainty of PTV dose and expand the possible gantry angles for coplanar IMRT beams. The beam attenuation while passing through the patient and the ability of the beams to fan out over more angles would be made possible by constraining beams to avoid the prosthesis, thereby causing a low dose to the prosthesis itself and the other normal tissue without compromising the target coverage. The drawback associated with this technique is related to the isocenter shifts to target implanted fiducial markers, which leads to the defined constraints for the beam meant for the implant shielding to be violated [51].

Another study employed the use of the IMRT-PAV (Prosthesis avoidance volumes) technique wherein the beam setup default without prosthesis in place is used as the starting point, and adjustments are made possible with the beam’s eye projection of the planning target volume (PTV) [52]. The beams could deposit the dose in the prosthetic device at the exit site of the PTV as dose delivery through the implant device at the entrance site of the PTV is prevented. This was achieved by defining a virtual organ-at-risk for each beam passing through the implant. These avoidance volumes needed to be placed outside the patient at minimal distances from the body contour and avoid overlaps with the beam’s eye view projections of other beams. The avoidance volumes were then delineated in the transverse images, conforming the view projections in front of the PTV with an additional 6 mm margin to account for the set-up uncertainties. The projection of each avoidance volume was evaluated after assigning each avoidance volume the electron density of water using quadratic overdose constraint for each avoidance volume and restricting the root mean square dose excess to 0.02 Gy. The results showed dose delivery to the surrounding bone around the prosthesis was higher, but the maximum dose was within constraints, and mean amounts to the bladder and rectum were reduced by up to 25%. The technique required more time for delineation, but the time for treatment planning was decreased due to applying the default beam setup.

The comparison of the coplanar and non-coplanar IMRT planning using beams that entirely avoided the implant but did not always cover the entire PTV showed that the non-coplanar plan had better rectum sparing; however, larger bladder volume was exposed to doses up to 50 Gy [53]. The study involved using 11 IMRT beams at three couch angles, resulting in a significantly longer treatment time than seven coplanar sets of treatment beams with gantry angles close to the beam directions that would be used without the prosthesis [52]. The use of Monte Carlo calculations was not found to be essential for obtaining the dose distributions as none of the photons that first traversed the prosthesis delivered the primary dose to the target or the organs at risk, and the difference in the dose delivered was within 2% or 2 mm in regions with high-dose gradients [54].

Another technique has been described that involved excluding beamlets from the optimization that would deliver the dose to the PTV while first passing through the implant [55], which led to the reduction in dose delivery to the bladder and rectum with an enhanced degree of freedom in selecting beamlets with predominantly lateral PTV irradiation. The rectal sparing was better achieved using 11–13 coplanar beams. However, there was a note of increased patient volume being treated to low doses and increased treatment times due to setting up times and each beam verification. Longer treatment times have been shown to impact the intrafraction motion [56], and thus, the treatment time could be reduced by utilizing nine coplanar beams with 10MV photon beams [55].

#### VMAT planning strategies with bilateral hip prosthesis

VMAT planning has become a standard of treatment delivery in prostate cancer [57, 58]. However, prosthesis avoidance is difficult due to several partial arcs needed for the beam arrangements. However, the delivery of treatment with the VMAT technique is possible with the use of the avoidance sector feature with the Varian linear accelerators, leading to the use of a complete arc and the beam shutting off temporarily at gantry angles that could deliver entry dose to the hip prosthesis [59, 60]. Another study utilized complete arcs but limited the hip prosthesis entry dose with an avoidance optimization structure around the implant with a 1 cm margin and a dose constraint of 500 cGy [61]. There has been a comparison of three different VMAT planning techniques, including two complete arcs with a maximum dose of 500 cGy to the prosthesis, two full arcs with a dose maximum of 500 cGy to 105 of the prosthesis, and six partial arcs avoiding the prosthesis and found improved dose-volume metrics for the rectum and bladder with us of the full arcs and significant improvement in the conformality, homogeneity and gradient indices favoring the use of the full arcs and improved dosimetric quality and deliverability with minimal entrance doses to each prosthesis [62].

#### Acuros XB algorithm and comparison with Monte Carlo calculations

The Acuros XB (AXB) algorithm by Varian Medical Systems, Inc. (VMS) (Palo Alto, CA) is a grid-based linear Boltzmann transport equation solver as a part of the Eclipse treatment planning system (TPS) (VMS) and was the first to help sharpen the dose gradients in the vicinity of the metallic implant [63, 64]. Studies have shown that the AXB algorithm can produce dose calculations with accuracy close to the Monte Carlo model [63], and the algorithm has been compared with the Monte Carlo regarding the lateral dose profile [64]. A comparative analysis of the use of the AXB on clinical patient plans showed the algorithm was reliable as a calculation algorithm in the case of planning in the presence of hip implants. It calculated the correct dose distribution at the metal-target interface. Thus, it helps avoid the need for the beams to traverse through the implant [65]. The authors also concluded that dose distribution calculations by the AXB algorithm showed minor deviations, on average within 2.5%, when compared to measurements and the MC model, but the performance was considered acceptable. An anisotropic analytical algorithm (AAA) was discouraged due to the uncertainty associated with the implant interface and inaccurate accounting of the backscatter due to the implant and the prosthesis-associated beam attenuation.

### Target localization during daily treatment

#### CBCT for daily image guidance

CBCT helps improve tumor targeting during delivery of the radiation treatment, helps reduce the dose to the organs at risk, and helps in the correct patient setup while monitoring with adequate soft-tissue visualization [66]. However, these series do not provide accurate Hounsfield units and do not have value for direct dose calculation [67]. However, with the hip prostheses in place, the CBCT images show much more scatter than the conventional fan-beam-based CT images [68]. Thus, Hounsfield unit calibration is required. It resulted in dose-calculation errors of > 5% compared with planning CT-based dose calculations when Hounsfield units-electron density conversion curves were based on average CBCT HU values for different treatment sites [69]. Other measures to adjust the Hounsfield unit include mapping these units in the CT images to equivalent points in the CBCT image geometry after deformable image registration (DIR) [70] or by using image cumulative histograms to adjust the units for the CBCT and the planning CT images [67]. The use of fat and muscles instead of water can also be employed and has been shown to have resulted in a dosimetric difference of < 2% [71] and has been referred to as density override. In one study, the CBCT voxels were assigned as water alone, followed by water or bone, then compared for scatter correction. The automated density override approach showed six different densities (air, lung, adipose, connective tissue, bone, and higher densities for prosthesis), and the CBCT image histograms were binned into six density levels. The comparison with the planning CT images showed superior outcomes in more minor patients with an anteroposterior diameter of less than 25 cm [72]. Previous studies have shown the need for a multilevel threshold algorithm to be helpful with single hip prosthesis and have helped reduce the operator time [73].

#### Electromagnetic localization tracking accuracy

The implantable electromagnetic transponders help continuously monitor the prostate location and help reduce the PTV margins, which helps reduce the doses to organs at risk, particularly the anterior rectal wall [74]. Generally, using these transponders has been considered a contraindication due to the interference of the radiofrequency signals of the transponders interfering with the prosthesis [75]. However, Bittner et al., in their case report, evaluated the reliability of positional data obtained through the tracking of the prostate phantom in the presence of a hip prosthesis and concluded that there was minimal effect on the ability to track these transponders [76] accurately. The maximum offset and the average offset recorded with the unilateral hip implant were 0.7 mm and 0.5 mm, and the bilateral implants were 0.5 mm and 0.3 mm, respectively.

#### Monitoring intrafraction motion

With the increased use of image-guided radiation therapy, the need for tumor motion management has been explored [77, 78] due to prostate motion associated with rectal and bladder filling differences. With the use of software related to commercial tracking systems, including Calypso (Varian Medical Systems, Palo Alto, CA) and Micropos (Micropos Medical AB, Goteborg, Sweden) [79–83], the implanted fiducials can be visualized and help assess the intrafraction motion. Also, the automated beam hold (ABH) provided with the TrueBeam linear accelerator (version 2.0 and onward, Varian Medical Systems) helps assess the kilovoltage images acquired during the beam on time. These images are the triggered images, which are obtained at predefined intervals during treatment delivery, and the implanted fiducials are automatically visualized [84]. The use of ABH accurately detects increased tumor motion, thereby increasing the prostate treatment delivery accuracy. Rosario et al. showed successful intrafraction motion detection with kV imaging, which helped reduce the PTV margins from 6 mm to 5 mm [84]. Similar results were seen with 3-dimension imaging using the Calypso system and function of time, and they showed that for treatments completed in 5 min (VMAT) and 10 min (IMRT), the proportion for the prostate to shift by > 3 mm was 4% and 12%, respectively [85]. The role is thus under evaluation and needs to be investigated further to incorporate the need for treatment planning for prostate SBRT in the presence of the hip prosthesis.

## Conclusion

In our review, we have presented the image-registration techniques, contouring, and planning aspects in patients with prostate cancer in the presence of hip implants. There is no available retrospective or prospective literature providing the clinical outcomes of SBRT in prostate cancer in the presence of hip implants, highlighting the fact that there is a hindrance in considering such patients for treatment with SBRT. The mentioned literature in this review looks into dosimetric aspects, and some studies have been conducted on the cadavers. Hence, the literature available is not robust enough to conclude whether the measures highlighted in the review can be implemented in the real-case scenario, and further prospective studies are needed to conclusively state whether such hip prosthesis is necessarily a contraindication for SBRT. With a rise in the number of patients undergoing hip replacement surgeries and the incidence of prostate cancer also rising, there is a need for better clarity on the aspects of SBRT treatment. There is a concern regarding the maximum dose in the bladder and the rectum, target delineation, image matching during the treatment, and transponders matching. However, the available reports encourage planning patients with this treatment modality. The review also highlights the need for future clinical trials to look into the survival and toxicity profiles on the impact of the prosthesis’s presence in prostate cancer management. Overall, there does not seem to be a need to consider the presence of hip implants as a contraindication for the SBRT treatment.

## Data Availability

Not applicable.
